# AIDing Chromatin and Transcription-Coupled Orchestration of Immunoglobulin Class-Switch Recombination

**DOI:** 10.3389/fimmu.2014.00120

**Published:** 2014-03-28

**Authors:** Bharat Vaidyanathan, Wei-Feng Yen, Joseph N. Pucella, Jayanta Chaudhuri

**Affiliations:** ^1^Weill Cornell Graduate School of Medical Sciences, New York, NY, USA; ^2^Immunology Program, Memorial Sloan Kettering Cancer Center, Gerstner Sloan Kettering Graduate School, New York, NY, USA

**Keywords:** cytidine deamination, DNA recombination, DNA repair, class-switching, R-loops

## Abstract

Secondary diversification of the antibody repertoire upon antigenic challenge, in the form of immunoglobulin heavy chain (IgH) class-switch recombination (CSR) endows mature, naïve B cells in peripheral lymphoid organs with a limitless ability to mount an optimal humoral immune response, thus expediting pathogen elimination. CSR replaces the default constant (C_H_) region exons (Cμ) of IgH with any of the downstream C_H_ exons (Cγ, Cε, or Cα), thereby altering effector functions of the antibody molecule. This process depends on, and is orchestrated by, activation-induced deaminase (AID), a DNA cytidine deaminase that acts on single-stranded DNA exposed during transcription of switch (S) region sequences at the IgH locus. DNA lesions thus generated are processed by components of several general DNA repair pathways to drive CSR. Given that AID can instigate DNA lesions and genomic instability, stringent checks are imposed that constrain and restrict its mutagenic potential. In this review, we will discuss how AID expression and substrate specificity and activity is rigorously enforced at the transcriptional, post-transcriptional, post-translational, and epigenetic levels, and how the DNA-damage response is choreographed with precision to permit targeted activity while limiting bystander catastrophe.

## Introduction

B cells are specialized lymphocytes that express Ig receptors (or antibodies) on their cell surface. Antibodies are comprised of immunoglobulin heavy chains (IgH) and light chains (IgL), with the N-termini of IgH and IgL generating the antigen-binding pocket, and the C-terminus of IgH performing effector functions. A salient feature of B-lymphocytes is their ability to recognize an almost infinite array of antigens. This enormous diversity is achieved through V(D)J recombination, a process that assembles the exons encoding the amino-terminal variable regions of IgH and IgL from component variable (V), diversity (D), and joining (J) segments (1). The end product of V(D)J recombination is a mature but naïve IgM^+^ B cell that exits the bone marrow. In the context of specialized structures called germinal centers in secondary lymphoid organs such as the spleen and lymph nodes, mature B cells interact with antigens and undergo class-switch recombination (CSR) (2, 3).

**Figure 1:**
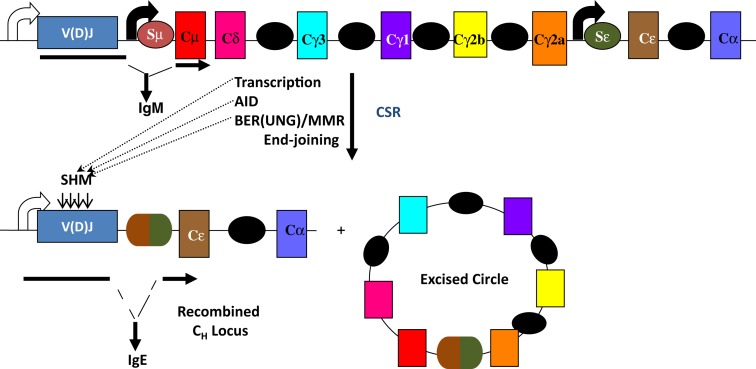
**Overview of CSR**. CSR is a deletional-recombination reaction between repetitive switch (S) regions (ovals) that precede each set of constant region (C_H_) exons. Cytokine-induced transcription through S regions drives CSR to specific C_H_ exons. CSR requires AID and components of the base-excision repair (BER) and mismatch repair (MMR) pathways. Somatic hypermutation (SHM) introduces point mutations at a very high rate into variable region exons, V(D)J.

**Figure 2:**
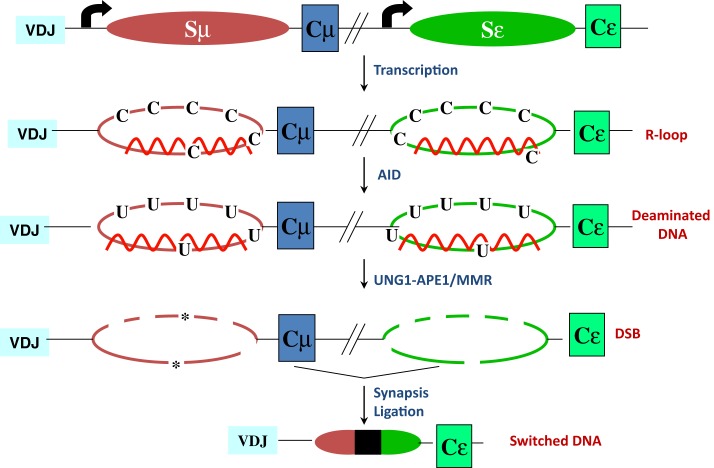
**R-loop-based model for CSR**. Transcription through S regions generates R-loop structures in which the RNA stably hybridizes to the template strand, displacing the non-template strand as ssDNA. AID activity at R-loops generate uridines in the DNA, which are subsequently processed by BER and MMR proteins to ultimately generate DSBs. End-joining of DSBs completes CSR. Asterisks represent nicks close to each other on opposite strands.

The mouse IgH locus is comprised of eight constant region (C_H_) exons, with Cμ most proximal to the variable region segments and Cα being the most distal (Figure 1). CSR exchanges the default Cμ for an alternative set of downstream C_H_ exons, for example, Cγ, Cε, or Cα, so that the B cell switches from expressing IgM to one producing a secondary antibody isotype such as IgG, IgE, or IgA, respectively. CSR occurs between repetitive “switch” (S) DNA elements that precede each set of C_H_ exons. According to the conventional model for CSR, transcription through S regions promotes formation of DNA:RNA hybrid structures, such as R-loops that reveal single-stranded DNA (ssDNA) substrates for activation-induced deaminase (AID)-mediated cytidine deamination (Figure 2). The deaminated residues are processed into DNA double-strand breaks (DSBs) by components of the base-excision repair (BER) and mismatch repair (MMR) pathways (4–6). End-joining of DSBs between two S regions results in the excision of the intervening sequence and juxtaposition of a new set of constant region exons directly downstream of the rearranged V(D)J segment, thereby generating Ig molecules with the same antigen specificity but with new effector functions (2, 3) (Figure 1). Here, we will discuss the intrinsic properties of AID, the factors commissioning its function to produce obligatory DNA DSBs intermediates, and the DNA repair/end-joining pathways that ensure productive recombination.

Germinal center B cells also undergo another AID-dependent secondary diversification reaction termed somatic hypermutation (SHM) wherein point mutations, and sometimes insertions and deletions, are introduced at a very high rate (10^−2^–10^−3^/bp/generation) into the recombined variable region exons encoding IgH and IgL, so as to select B cells with increased antigen affinity (Figure 1). SHM requires transcription through the variable region exons and occurs primarily, but not exclusively, at RGYW “hot-spot” motifs where R = purine base, Y = pyrimidine base, and W = A or T nucleotide. Details of SHM are outside the scope of this review and have been discussed in multiple excellent reviews [for example, Ref. (7)].

## Intrinsic Properties of AID

Following the discovery of AID by subtractive cDNA hybridization from the mouse CH12F3 B lymphoma cells and its proven essentiality for SHM and CSR (8, 9), a huge amount of effort has gone into the characterization of its enzymatic properties. Elegant biochemical work using purified AID from activated splenic B cells, recombinant GST-AID from Sf9 cells, and other epitope-tagged forms, has shed light into the DNA deamination ability of AID *in vitro* [reviewed in Ref. (2)]. These studies demonstrated unequivocally that AID deaminates deoxycytidines (dCs) in ssDNA, and fails to act on dsDNA, RNA, and DNA:RNA hybrids. Additionally, it was shown that AID could deaminate dCs in the context of transcribed dsDNA (10, 11), suggesting that access to and activity on *in vivo* substrates might require transcription of the locus. Since the crystal structure of AID has not been determined, the field has faced a bottleneck in explicit elucidation of enzyme biochemistry. Still, based on structural and biochemical insights from bacterial cytidine deaminases and related DNA/RNA deaminases such as APOBECs, the mechanism of Zn^2+^-dependent catalysis by the active site (H56, E58, C87, C90) residues and preference for RGYW motif (residues 113–123) was cogently demonstrated (12, 13).

*In vitro* deamination assays using recombinant GST-AID purified from insect cells suggest that AID performs processive catalysis (14), which leads to accumulation of multiple mutations on a single DNA fragment, disfavoring “jumping” onto a second fragment. This finding is in contrast to proposed distributive mode of action based on the high net positive charge (+11 at pH 7.0) of AID that promotes strong binding to nucleic acids (15). Nonetheless, *in vitro* deamination assays performed on ssDNA substrates revealed that AID-mediated deamination is intrinsically inefficient, haphazard, and a “random bidirectional walk” along DNA, yielding ~3% deamination upon hot-spot encounter (16). Such a mechanism has likely evolved to generate a diverse array of mutations, especially to favor the selection of high affinity antibodies *in vivo* (16). A note of caution to be borne in mind while interpreting these biochemical analyses is that the bulky tag might affect inherent properties of AID, and GST-AID does not reconstitute CSR *in vivo* (17), suggesting that *in vitro* results may not accurately reflect the *in vivo* scenario. Besides, this form of AID requires the action of RNase A to be active *in vitro*, which contradicts reports of AID purified from B cells (10), advocating for adventitious properties unique to GST-AID. It is to be noted that AID, based on its homology to the RNA editing enzyme APOBEC1, has been proposed to edit mRNAs and/or micro-RNAs (miRs) required for CSR and SHM, however, there is no experimental evidence yet to support this notion (18). Thus, despite the limitations of AID enzyme biology, strong genetic evidence has propelled DNA deamination as being the generally accepted model, and this review is based on this premise.

## Regulation of AID Expression, Localization, and Stability

While the primary and physiological role of AID is to introduce DNA lesions at the Ig loci to drive antibody diversification, AID also poses a threat to genomic integrity. Ectopic expression of AID in non-B cells converts it into a general mutator (19, 20). Even in B cells, mistargeted AID activity is the major underlying cause behind oncogenic translocations that are hallmarks of a large number of B cell malignancies (1, 21). Therefore, regulation of AID expression is fundamental not only for the development of an efficient immune system, but also for the maintenance of genomic integrity inherent to cells expressing a mutator. Thus, it is not surprising that AID comes outfitted with multifaceted transcriptional and post-transcriptional regulatory mechanisms.

### Transcriptional regulation of AID expression

**Figure 3:**
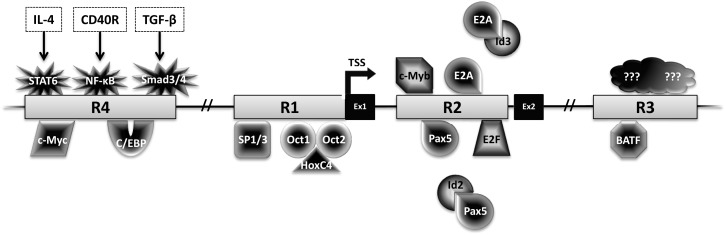
**Transcriptional regulation of AID**. The *Aicda* locus contains four conserved regulatory regions (R1–4). The first two exons (Ex1–2) and transcription factors with the potential to bind these regions are shown. Factors with black center and white text are activators, while factors with white center and black text are repressors.

Activation-induced deaminase is encoded by the *Aicda* gene, located on chromosome 6 and 12 in mice and humans, respectively. Four highly conserved regulatory regions activate *Aicda* transcription primarily in activated B cells, and restrict its expression in other cell types (Figure 3). Region 1 is comprised of a TATA-less promoter and enhancer elements that bind HoxC4–Oct1/2 and Sp1/3 [reviewed in Ref. (22)]. This region also contains elements that respond to estrogen and progesterone, hormones that, respectively, activate or repress AID expression (23–25). Region 2 lies within the first *Aicda* intron and includes binding sites for B-cell-specific Pax5 and E2A proteins (22). This region also harbors silencer elements that could bind repressors E2F and c-Myb in a fashion unrestricted to B cells (22). Deletion of the silencer elements drastically increases AID expression, without inducing transcription in non-B cells, bolstering the notion of extensive checks to AID expression (26). Region 3, approximately 25 kb downstream of *Aicda*, is necessary to sustain physiological levels of AID expression, likely through a BATF-binding site (22, 27, 28). Region 4 is approximately 8 kb upstream of the *Aicda* transcriptional initiation site and contains enhancers that bind NF-κB, STAT6, and SMAD3/4, factors that are stimulated by B cell activation (22). Recently, c-Myc was implicated in binding Region 4 to promote robust AID expression (29, 30).

Although physiological AID expression is largely restricted to mature B cells, its expression has also been reported in other settings. AID is expressed in developing B cells in the bone marrow, inducing robust CSR to a subset of isotypes (31, 32). The physiological relevance of CSR in the bone marrow is not clear at present. AID expression has also been observed in intestinal epithelial cells during *Helicobacter pylori* infection; whether this represents aberrant expression or some uncharacterized response to infection is not known (33, 34). Additionally, AID expression has been observed in prostate cancer cells (35); such aberrant AID expression might be correlative or causal to pathological outcomes.

It is not clear at present whether the non-B-cell-specific expression of AID has any physiological relevance, and the AID fate-mapping mouse does not reveal robust expression pattern in non-lymphoid cells (36). But an intriguing finding is that AID is expressed in primordial germ cells, in embryonic stem (ES) cells, and also in mouse embryonic fibroblasts induced to undergo transcription-factor-mediated reprograming (37, 38). In this regard, AID has been posited to deaminate methylated cytidine, and in concert with DNA BER, promote demethylation of genes required for the maintenance of a pluripotent stem-cell state (37–40). However, AID has extremely weak intrinsic activity on 5mC, and AID-deficient mice do not exhibit any overt phenotype or methylome changes that could be attributed to a failure in active demethylation (41, 42). Thus, *in vivo* demethylation by AID and the factors regulating AID expression in these settings remain provocative and warrant further research.

### Micro-RNA-mediated regulation of AID expression

Another level of regulation exists at the level of stability of *Aicda* mRNA, enforced by miRs such as miR-155, miR-181b, and miR-361, with miR-155 being the best characterized. miR-155 expression is upregulated upon activation for CSR. The 3′-untranslated region (3′-UTR) of *Aicda* mRNA has a binding site for miR-155, mutation of which, increased AID expression and doubled the frequency of CSR (43, 44). Surprisingly, although miR-155-deficient B cells upregulate AID expression, they do not undergo increased CSR, perhaps due to dysregulation of other miR-155 targets relevant to CSR (45). The 3′-UTR of *Aicda* mRNA also contains a binding site for miR-361 (46). Significantly, the transcription factor Bcl6, required for formation of germinal centers, binds and transcriptionally represses both miR-155 and miR-361, in turn relieving repression of AID (46). The role, if any, of miR-361 in the regulation of AID mRNA stability remains to be determined. Finally, ectopic expression of miR-181b in activated murine B cells impaired CSR, likely due to reduced AID mRNA and protein levels (47). Given the emerging significance of canonical and non-canonical miR targeting, it can be conceived that many more miRs affecting AID and CSR are awaiting discovery (48).

### Subcellular localization and stability

**Figure 4:**
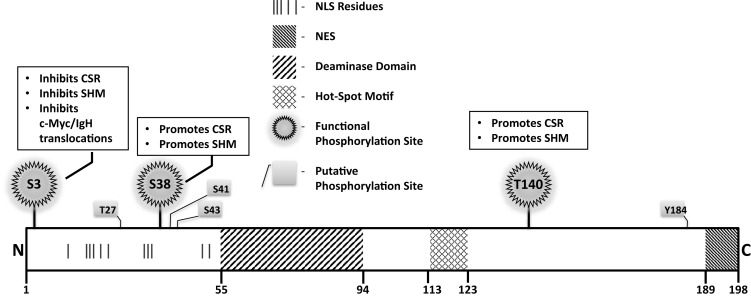
**Notable AID domains and residues**. Primary structure of AID protein with pertinent domains, motifs, and residues indicated. Panels outlined in black briefly describe functional impact of validated phosphorylation sites.

A rational way to constrain AID activity on DNA is by regulating its subcellular localization. AID localization is governed by active nuclear import, cytoplasmic retention, and efficient nuclear export. The majority (greater than 90%) of AID is sequestered in the cytoplasm, possibly through interactions of the C-terminus of AID with eEF1A, chaperone Hsp90, and co-chaperone Hsp40 DnaJa1 (49). Nuclear entry of AID is dependent on importin-3 and a conformational nuclear localization signal (NLS) generated upon folding; a predicted bipartite NLS at the N-terminus of AID might not be functional (49) (Figure 4). In the nucleus, AID was recently found to accumulate in nucleolar structures where it associates with nucleolin and nucleophosmin (50). Mutations that abrogated AID localization to these structures resulted in reduced levels of CSR (50). The nucleoli may serve as a nucleation site for forming complexes, but the precise role of nucleolar AID remains unresolved.

A mutator protein’s presence in the nucleus must be vigilantly regulated, and a nuclear export signal (NES) within the last 10 amino acids at the C-terminus of AID mediates CRM1-dependent active nuclear export (49) (Figure 4). Mutations in the NES increased levels of nuclear AID, enhanced SHM, but severely impaired CSR, indicating that NES-bearing C-terminus of AID plays a role in CSR beyond export, perhaps in mediating CSR-specific interactions (49). Consistent with this notion, replacement of the C-terminus of AID with a heterologous NES rescued nuclear export, but did not reconstitute CSR (51). Strikingly, the stability of AID was often compromised upon manipulation of the C-terminus of AID, even when nuclear export remained unaffected (51). In this regard, the half-life of nuclear AID is significantly shorter than its cytoplasmic counterpart (~2.5 vs. ~18 h) (52). This is largely due to interactions of AID with the proteasome through ubiquitination or Reg-γ-mediated escort (52, 53). Overall, the involvement of the C-terminus of AID in mediating nuclear export, protein stability, cytoplasmic retention, and CSR-specific interactions render this region one of the most fascinating, yet complicated domain that demands extensive examination.

### AID phosphorylation

Numerous putative phosphorylation sites in AID have been implicated in regulating its ability to effect CSR, SHM, and oncogenic translocations, without affecting stability or deamination potential (Figure 4). Unfortunately, for most, mechanistic insights of functional pertinence remain elusive. Physiologically relevant sites that play critical to modest roles in AID function include serine-3 (S3), threonine-140 (T140), and S38 (54–58). Serine-3 was identified as a site phosphorylated by protein kinase C (PKC) *in vitro* (55). In contrast to other validated phosphorylation events, phosphorylated S3 inhibits AID function. Mutation of S3 to alanine enhances CSR, SHM, and *c-Myc/IgH* translocations, despite unperturbed catalytic activity (55); however, the mechanistic underpinnings remain unresolved. PKC can also phosphorylate T140, and T140A mutation perturbs SHM more profoundly than CSR. The mechanism through which phosphorylation at T140 differentially regulates SHM and CSR remains unclear (56). Phosphorylation of AID at serine-38 has been extensively characterized and will be discussed later. Overall, the regulatory mechanisms discussed above, and processes that mediate substrate specificity *in vivo* as discussed below, impose checkpoints in maintaining physiological functions of AID to facilitate successful and efficient CSR.

## Accessibility and Targeting of AID

Since AID is an ssDNA deaminase, mechanisms must exist to generate and reveal such structures at S regions during CSR. Additionally, AID must be actively and specifically recruited to S regions, not only to be productively engaged in CSR, but also to reduce collateral damage associated with expression of a mutator protein. The nature of S regions and their transcription promote AID accessibility to DNA while several proteins have been implicated to specifically recruit AID to the IgH locus during CSR.

### S regions, transcription, and R-loops

S regions are 1–12 kb repetitive sequences that are enriched with AID “hot-spot” 5′-RGYW-3′ motifs (59, 60), and are particularly G-rich on the non-template strand. Evidence for the role of S regions came from elegant genetic studies wherein deletion of Sμ dramatically impaired CSR to all isotypes while deletion of Sγ1 abolished CSR to IgG1 (61–63). Recent studies have provocatively suggested that apart from the default donor Sμ, even Sγ1 can serve as a donor and allow sequential switching to IgE (64), an idea that was suggested two decades back when double-isotype expressing B cells were identified (65–68).

**Figure 5:**
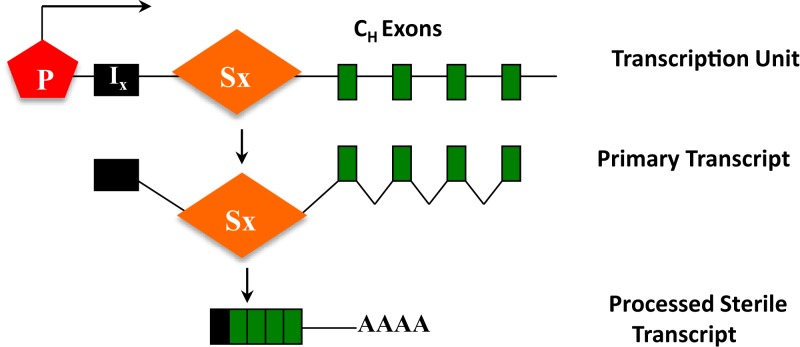
**C_H_ exons as transcription units**. Each set of C_H_ exons is an independent transcription unit comprised of a promoter (P), an intervening I-exon, an intronic-switch (S) region, and C_H_ exons. The primary transcript is spliced and polyadenylated but does not have protein-coding capacity, and is thus referred to as a sterile transcript.

The ability of S regions to serve as recombination targets is intricately linked with “germ-line” transcription, an essential prerequisite for CSR (2, 3). Each set of C_H_ exons is an independent transcriptional unit, comprised of an intervening (I)-exon, intronic S region, and the C_H_ exons (Figure 5). The primary transcripts produced constitutively (via μ promoter) or inducibly (for other C_H_ exons), are spliced and polyadenylated but have no protein-coding capacity. These are referred to as germ-line or sterile transcripts. Differential stimulation with distinct sets of activators and cytokines, provided by helper-T cells or through direct interaction with pathogens, induces transcription through different C_H_ exons and promotes CSR to that particular isotype. Significant progress in our understanding of CSR came from *ex vivo* studies wherein splenic B cells were activated in culture under different conditions. For example, bacterial lipopolysaccharide (LPS) induces germ-line transcription through Cγ2b and Cγ3 and allows CSR to IgG2b and IgG3, while a combination of LPS and interleukin-4 induces Cγ1 and Cε transcription and CSR to IgG1 and IgE. Mutational analyses that altered or deleted the I-exon promoter perturbed CSR dramatically, thus providing experimental evidence for the strong mechanistic link between germ-line transcription and fulfillment of CSR (2, 3).

It is generally believed that transcription through S region sequences promotes formation of R-loops, wherein the template strand stably hybridizes with the G-rich primary transcript (69, 70). This allows the non-template strand to be looped out as ssDNA, providing an ideal substrate for AID (2) (Figure 2). Compelling work in support of the R-loop model came from the observations that a transcribed synthetic DNA fragment with a G-rich non-template strand can support AID deamination *in vitro* and CSR in B cells, while the inverted sequence (C-rich non-template strand) that does not form R-loops, neither supports AID-mediated deamination *in vitro* nor CSR *in vivo* (10, 63). It is to be noted that although the role for germ-line transcription has been well-studied, a possible role of the transcript *per se* was suggested from the observation that perturbing splicing of primary switch transcripts without affecting transcription impedes CSR (71, 72). However, the neomycin-resistance cassette used in targeting the splice donor site was not removed, leaving open the possibility that the observed CSR defect was due to non-specific effects of this cassette in the IgH locus. Despite this potential caveat, given how non-coding RNAs like HOTAIR and Xist drive PRC2 targeting (73, 74), it would not be surprising if these non-coding switch transcripts play a significant role in AID targeting and activity at S regions.

### Factors promoting template strand deamination

The R-loop model does not account for the mechanism of template strand deamination by AID, a prerequisite for the formation of DSBs. Several models have been put forward to account for deamination of template strand. Anti-sense transcription through the IgH locus has been proposed to facilitate access of AID to the template strand (75); however, anti-sense transcription is not essential for CSR (76). Components of the RNA exosome complex have been shown to interact with AID and mediate accessibility to the template strand by degrading the nascent RNA hybridized to the template strand (77). Recent work cogently elucidated that Nedd4-dependent ubiquitination modulates the fate of AID-associated RNA polymerase II (Pol II), thus generating free 3′-ends that serve as substrates for RNA exosomes (78). RNaseH has also been proposed to facilitate R-loop collapse to ensure template strand deamination (79). However, the kinetics of such R-loop degradation must be stringently regulated in the context of S regions to first allow AID to act on the non-template strand, and elucidation of such intricacies awaits future work.

### Targeting AID to DNA

The primary sequence of S regions, transcription, and R-loops set a platform favorable for AID activity. However, for AID to reach this platform inside the nucleus is analogous to finding a needle in a haystack. Although AID-instigated off-target breaks are incurred, the frequency is far less than what is observed for the Ig loci (80–82). The low abundance of AID at the non-Ig genes has led to the debate whether this represents true binding or mere background creeping into the chromatin immunoprecipitation-sequencing (ChIP-seq) analysis used in these experiments (83). While genome-wide occupancy studies suggested that AID associates with accessible chromatin at stalled promoters of transcribed genes (82), reanalysis of the same data set (83) contradicted the notion of genome-wide AID binding. The technicalities and subtleties of data normalization for ChIP-Seq studies seem to be at the heart of such disparate results, and do highlight the need for caution when interrogating chromatin binding of proteins with low nuclear abundance (83). Thus, while both genome-wide (82) and locus-specific ChIP (10, 81, 84) clearly show abundance of AID at the Ig loci, the efficiency of its binding to other genomic sequences needs to be re-evaluated. Nonetheless, AID-induced mutations at non-Ig genes are observed in even normal B cells (80). Thus, it is obvious that the process is stringently orchestrated to prevent bystander damage by AID. Several elements within the Ig loci have been implicated in targeting AID to the variable region exons during SHM (85); however, in this review we will primarily describe the factors that chaperone AID with exquisite precision to the S regions during CSR.

Activation-induced deaminase was shown to be in a complex with Pol II (84), and more specifically with Spt5, a Pol II-associated protein mechanistically linked to transcriptional pausing (81). Genome-wide Spt5 occupancy correlated significantly with stalled Pol II and was predictive of AID-dependent mutations. B cells depleted of Spt5 had a severe defect in CSR, a consequence of decreased AID binding to S regions (81). A comprehensive treatise on the role of RNA pol II pausing at S regions during CSR has been reviewed elsewhere (86). The germinal center-specific GANP protein has also been implicated in mediating AID–Spt5–Pol II interaction (87). However, GANP deficiency does not impair CSR. Thus, in the context of switching B cells, there might be other unidentified players that facilitate AID targeting to stalled Pol II, and recent studies have shown that members of the Pol II-associated factor 1 (PAF1) complex and histone chaperone FACT complex can promote immune diversification by regulating association of AID with Pol II (88).

The 14-3-3 adaptor proteins have been implicated in recruiting AID to DNA through their ability to interact with RGYW sequences (89). It is unclear and somewhat counterintuitive as to what happens to 14-3-3 proteins after they chaperone AID to DNA, and why they do not compete directly with AID for DNA binding. Additionally, recent data suggests that 14-3-3 proteins perform scaffolding function by directly interacting with uracil DNA glycosylase (UNG) and protein kinase A (PKA), two proteins with well-established functions in CSR (90–92). Besides, the data implicate an AID C-terminus-dependent complex formation with 14-3-3 and subsequent targeting, but they fail to reconcile how an AID Δ189–198 mutant that is impaired in 14-3-3 binding, can be targeted to the S regions and generate mutations (93). Future work is warranted to unequivocally establish the role of the 14-3-3 adaptors in AID targeting.

Polypyrimidine-tract-binding protein-2 (PTBP2) was identified as an AID interactor that regulates AID targeting to S regions (94). Originally known to be a splicing regulator in brain (neuronal isoform, nPTB), this protein also interacted with both the sense and anti-sense S region transcripts in primary B cells undergoing CSR. Since splicing might be important for CSR (71), it is tempting to speculate a splicing regulation-associated function of PTBP2 in AID recruitment to S regions. Molecular insights into PTBP2-dependent regulation of AID targeting, and the fate of nuclear AID in the absence of PTBP2 will surely constitute the next phase of investigation.

### Role of chromatin modifications in targeting AID

It is becoming increasingly clear that epigenetic marks play crucial roles in mediating S region accessibility (95, 96). Both donor and acceptor S regions are specifically enriched for acetylation and methylation marks at histones H3 and H4, generally associated with “open” chromatin, for example, H3K9/K14ac, H3K27ac, H4K8ac, and H3K4me3. It has been suggested that AID targeting to Sμ is facilitated by the H3K9me3 mark, which tethers AID to the donor S region via the HP1–KAP1 complex (97). Additionally, PTIP, a component of the mixed-lineage leukemia-like complexes that are important regulators of H3K4 methylation, participates in CSR by regulating transcription-coupled chromatin accessibility. PTIP-deficient B cells have a severe defect in CSR due to decreased germ-line transcription of downstream C_H_ exons, and compromised DNA repair (98). Finally, combinatorial H3K9ac and H3S10 phosphorylation (H3K9acS10ph), specifically in the recombining S regions, deposited by GCN5/PCAF in stimulated B cells, leads to 14-3-3 adaptor-dependent AID binding to permit efficient CSR (96). However, it is to be noted that these chromatin marks are not likely to be unique to S regions, and thus cannot be sole determinants of regions permissive to AID activity. Interestingly, it has been shown that R-loops are tightly linked to H3S10ph, a chromatin condensation signature (99). Thus, it can be posited that R-loop formation facilitates H3S10ph chromatin modification, which in combination with H3K9me3 and H3K9ac marks, permits AID-mediated *in vivo* deamination of S region targets. The precise interplay of chromatin “writers,” “erasers,” and “readers” that regulate these events warrants further investigation, but it is unambiguous that this complex recombination reaction must be impeccably tuned by such epigenetic controls to prevent collateral damage by AID.

## Generation of Double-Strand Breaks Downstream of DNA Deamination

All the regulatory mechanisms alluded to above serve to generate AID-instigated dU lesions in S regions. Since CSR proceeds through DSB intermediates, the deaminated S regions need to be processed into DNA nicks, with two closely opposed nicks constituting a DSB (2, 3). This is achieved by components of the BER and MMR pathways.

### Role of BER and MMR pathways

According to the prevailing model for CSR, UNG, a component of the BER pathway, removes the uracil base from deaminated S regions. The abasic site thus generated is converted into a nick by the apurinic/apyrimidinic endonuclease APE1. Two closely spaced nicks on opposite strands constitute a staggered DSB, further processing of which by nucleases or DNA polymerases (fill-in) generates a blunt DSB that can participate in end-joining (4, 5, 100). Consistent with this model, mutations in UNG lead to a severe defect in CSR, likely as a consequence of impaired formation of DSBs in S regions [reviewed in Ref. (2, 3)]. Additionally, APE1^±^ mice and APE1-deficient CH12 cells reflected decreased DSBs in S regions and compromised CSR (101, 102). Components of MMR pathway have also been demonstrated to process DNA during CSR through the ability of Msh2:Msh6 to bind dU:dG mismatches, and subsequently recruit exonuclease 1 (exo1) to potentially process nicks and ssDNA gaps into DSBs. Indeed, mutations in Msh2 and Exo1 alter S region junctions and significantly impair CSR [reviewed in Ref. (2, 3)]. Conversely, deficiency of Pms2 and Mlh1, other members of the MMR machinery, lead to increased microhomology at S region junctions, suggesting that they might act to suppress alternative end-joining (103, 104). However, UNG mutations have a more profound effect on CSR than mutations in MMR proteins, suggesting that CSR is more reliant on the UNG-dependent steps. Whether this reflects an uncharacterized preference for one pathway over the other or supports a proposed non-canonical role for UNG, independent of uracil removal activity during CSR, remains an open question (105).

### Generation of high density of DSBs: Requirement of AID phosphorylation at Serine-38

The cellular DNA end-joining machinery is highly efficient and it is conceivable that a single DSB at an S region will be repaired before it can synapse with and ligate to a downstream DSB (106). It has therefore been speculated that efficient CSR would require a high density of DSBs at S regions to promote productive long distance synapsis and recombination between acceptor and donor S regions over intra-switch re-ligation (92), a phenomenon commonly observed in B cells that have initiated CSR but failed to complete the process (2). Recent studies have suggested that AID phosphorylated at serine-38 (S38) by PKA interacts with APE1 to actively generate a high density of breaks, a likely prerequisite for CSR (92, 107). In keeping with this notion, mutation of S38 to alanine severely impairs CSR due to a failure to efficiently generate DSBs at S regions (54, 56–58, 92, 107, 108).

**Figure 6:**
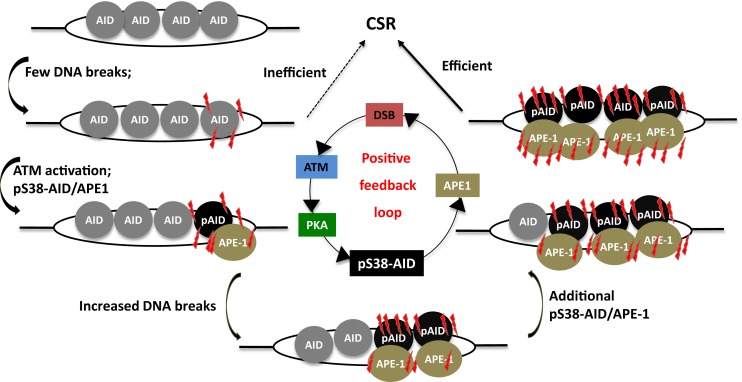
**A phosphorylation-dependent positive feedback loop drives CSR**. Assembly of AID at S regions induces low-density DSBs and leads to inefficient CSR, but ATM activation. This results in PKA-dependent AID phosphorylation at serine-38, which promotes interaction of AID with APE1. Active recruitment of APE1 to DNA accentuates DSB formation, which in turn induces phosphorylation of additional molecules of AID, thereby perpetuating the DSB amplification loop. This promotes the generation of high density of DSBs at S regions that is required for CSR.

Strikingly, AID phosphorylation at S38 was stimulated by DSBs (107). Thus, AID phosphorylation at S38 is both required for, and dependent on DSBs. This suggests the existence of a positive feedback loop wherein a low density of DSBs leads to AID phosphorylation, APE1 binding, and amplification of DSBs that feedback into the loop (Figure 6). It was also demonstrated that ATM, a protein critical for cellular response to DNA-damage, participates in sensing the DSBs at S regions, thereby promoting AID phosphorylation and APE1 interaction (107). Being a master regulator of the DNA-damage response, it is possible to envision ATM as a molecular rheostat that fine tunes DSB formation with efficient repair/recombination and allows safeguarded CSR while minimizing translocations. This is reminiscent of the role of RAG proteins in orchestrating V(D)J recombination by generating DSBs and efficiently channeling them to productive recombination, keeping translocation risks at bay (109–111). Similar to RAG-dependent coordination of break induction and repair, AID phosphorylated at S38 not only facilitates break formation, but also interacts with the ssDNA-binding protein, replication protein A, and likely enforces DNA repair pathways during CSR (112, 113).

Phosphorylation at S38 actively integrates AID functions into steps downstream of DNA deamination; however, several key questions remain elusive. First, the factors that facilitate APE1 binding to pS38AID need to be identified. Second, the regulatory mechanisms that couple chromatin sensing to DNA-damage signaling remain a mystery. Based on the recent finding of KAT5 (TIP60) tyrosine phosphorylation by DNA-damage to facilitate H3K9me3 binding and subsequent acetylation of ATM (114), it can be conjectured that such a pathway might be involved in the context of S regions and CSR, where the H3K9me3 mark has been shown to play a vital role (97). Finally, the steps between ATM activation and PKA-dependent AID phosphorylation remain a black box. These questions remain an active area of investigation.

## Completion of CSR: End-Joining of Switch-Region DSBs

Double-strand breaks generated at two distinct S regions are synapsed and ligated by end-joining during the completion phase of CSR (115). Below, we discuss the DSB response and DNA end-joining pathways that participate in this process.

### DSB response during CSR

**Figure 7:**
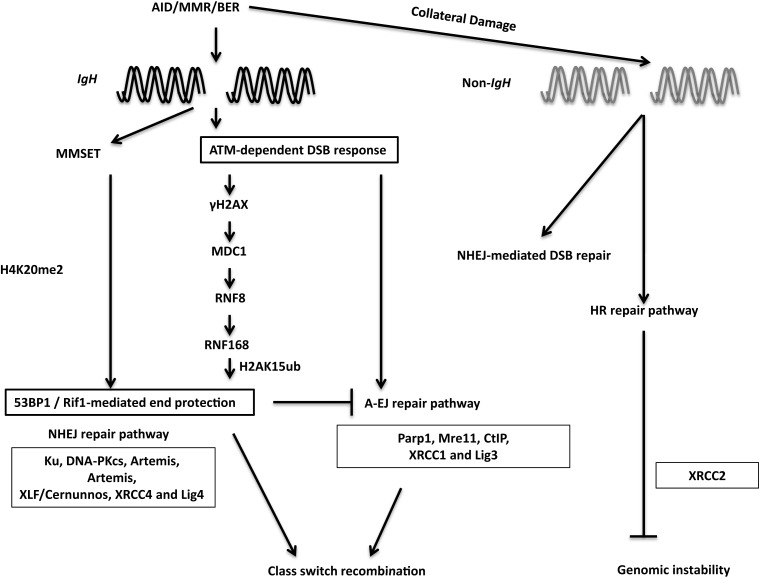
**DSB response during CSR**. The combination of AID, BER, and MMR proteins generate DSBs physiologically at the IgH locus and aberrantly at non-Ig regions, leading to activation of the cellular DNA-damage response pathways. 53BP1 is recruited to DSBs by directly binding to H4K20me2 or through the γH2AX–RNF8–RNF168 node (see text for details). 53BP1-dependent end protection facilitates ligation of two S regions by NHEJ and/or A-EJ. Collateral damage induced by AID at non-Ig genes is repaired by NHEJ and by XRCC2-dependent homologous recombination.

During the general DNA-damage response, DSBs are rapidly recognized by the Mre11–Rad50–Nbs1 (MRN) complex (116) (Figure 7). Nbs1 recruits and activates ATM, which phosphorylates H2AX. Phosphorylated H2AX (γH2AX) serves as a docking site for several DNA response proteins and promotes the rapid accumulation of 53BP1, Nbs1, and MDC1 into repair foci near DSBs (116). Deletion or mutation of Nbs1, H2AX, 53BP1, and ATM impaired CSR, indicating that the proteins that participate in sensing and transducing DSBs participate in CSR (116). Additionally, the ATM-dependent DNA-damage response is required for maintenance of genomic integrity and suppression of oncogenic translocations, possibly through enforcing cell-cycle checkpoints (116). Overall, ATM promotes the assembly of macromolecular foci that stabilize DNA ends and facilitate the recruitment of repair factors to ensure productive CSR while preventing oncogenic translocations.

Among the ATM-activated DSB response factors, 53BP1 deficiency leads to the most pronounced defect in CSR (116). CSR requires “synapsis” or close juxtaposition of donor and acceptor S regions (115, 117) and 53BP1 has been proposed to promote the synapsis of broken S regions during CSR (118). Furthermore, 53BP1 has been shown to associate with Rap1-interacting factor 1 (Rif1) to protect broken DNA ends from resection. Absence of Rif1 in B cells leads to increased DNA end resection, virtually phenocopying 53BP1 deficiency and providing functional significance to the 53BP1–Rif1 interaction during CSR (119–121). A recent study elegantly teased apart differential roles of distinct phosphoprotein interactions of 53BP1, and convincingly illustrated that Rif1 serves as an effector of productive repair, whereas PTIP wards against mutagenic repair. This study clearly demonstrated that 53BP1 is a key player at the crossroads of efficient/aberrant DNA repair pathway choice (122).

The chromatin microenvironment strongly influences the DSB response. Two mechanisms have been proposed to regulate 53BP1 recruitment to DSBs in the context of chromatin. The first relies on its interaction with H4K20me2. Methylation of H4K20 and subsequent 53BP1 recruitment to sites of DNA-damage is regulated by MMSET, a histone methyltransferase (123, 124). MMSET depletion in the CH12F3 B cell line decreases H4K20me2 levels, attenuates 53BP1 accumulation at S regions, and impairs CSR (123). The other process that recruits 53BP1 to DSBs requires the RING finger protein 8 and 168 (RNF8 and RNF168)-dependent histone ubiquitination pathway. RNF8 is recruited to ATM-phosphorylated MDC1 bound to γH2AX at the site of DSBs and catalyzes ubiquitin-dependent recruitment of RNF168 to chromatin flanking the DSBs (125). Recently, 53BP1 has been shown to recognize DNA-damage-induced H2A Lys15 ubiquitination catalyzed by RNF168, revealing the mechanism of RNF8/168-dependent recruitment of 53BP1 at DSBs. RNF8 deficiency compromised recruitment of 53BP1 to S regions in activated B cells and significantly impaired CSR. Additionally, inactivation of RNF168 impaired CSR in mice (126–129). Taken together, these observations suggest that 53BP1 recruitment plays a critical DSB end-protecting role during CSR.

### DNA end-joining

Non-homologous end-joining (NHEJ) is the primary mechanism used for end-joining during CSR (2). The canonical NHEJ machinery includes the Ku70/Ku80 heterodimer (Ku), DNA-dependent protein kinase catalytic subunit (DNA-PKcs), Artemis, XRCC4-like factor (XLF or Cernunnos), XRCC4, and DNA ligase IV (Lig4). Mutations in NHEJ components including Ku70/80, XRCC4, and DNA ligase IV severely compromise CSR. The role of NHEJ proteins in CSR is also evident from the observations that mutations in DNA-PKcs, artemis, and XLF lead to high levels of chromosomal IgH breaks and translocations, even in instances where CSR frequency is not severely impacted (116). Our current knowledge however, does not uncover how the initial recognition of DSBs by the MRN complex leads to the binding of Ku and DNA-PKcs to the broken DNA.

Non-homologous end-joining-deficiency does not abolish CSR, and S junctions in NHEJ-deficient B cells reveal extended microhomology, leading to the proposal that an alternative end-joining process (A-EJ) ligates DSBs during CSR (116). No factors unique to A-EJ have yet been characterized; several proteins involved in other DNA repair pathways have been implicated to function in A-EJ, including Mre11 and CtIP. Mre11 and CtIP have been implicated to trim broken DNA ends to uncover microhomology regions, generating short stretches of complementary nucleotides at DNA breaks, thereby promoting A-EJ during CSR (116). In CH12F3 cells, CtIP depletion impaired CSR to IgA and reduced the overall length of microhomology at the S junctions (130, 131). Notably, CtIP-deficient B cells undergo normal CSR to IgG1 (132). Therefore, elucidation of the role of CtIP in CSR requires further investigation. A major open question relates to the interplay between NHEJ and A-EJ: does A-EJ operate in presence of intact NHEJ and does it have a physiological role other than being a mere backup to NHEJ?

### Role of homology-dependent repair in resolving AID-induced breaks during CSR

It has been reported that AID instigates formation of widespread DSBs throughout the genome in activated B cells, albeit at significantly lower levels than that at the IgH locus (133–136). Such off-target DSBs at non-IgH loci are the major underlying lesions contributing to translocations between IgH and non-IgH loci (such as *c-Myc*) in B cells and are largely responsible for the ontogeny of a large number of B cell lymphomas in humans (21). In addition to aberrant translocations, AID can also induce somatic mutations at numerous loci linked to B cell tumorigenesis (80). While AID-initiated DSBs are observed in the G1 phase of the cell cycle (137), and CSR is likely completed before the cells transit into the S phase, it has been suggested that homologous recombination (HR)-dependent repair has a major role in providing resistance to AID-induced off-target DNA-damage. This is based on the observation that B cells deficient in the HR protein XRCC2 have significantly enhanced AID-dependent genome-wide DSBs (138) (Figure 7). Notably, the interplay between AID-mediated DNA breaks and HR repair pathway has been used in clinically relevant studies wherein AID-expressing human chronic lymphocytic leukemia cells were shown to be hypersensitive to HR inhibitors, possibly due to AID-dependent synthetic cytotoxicity (139). Further studies, in clinical settings, should be an interesting and possibly efficacious way to turn the mutator activity of AID into a therapy for B cell malignancies.

## Perspective

The discovery of AID was a watershed event in the field of B cell biology and in deciphering the underlying cause behind the ontogeny of a large number of B cell lymphomas. We now have a working model of how non-coding transcription and DNA deamination initiate CSR and how general DNA repair proteins that function in distinct pathways contribute to the process. Still, a large number of unknowns plague the field. These include the mechanisms that specifically recruit AID to the Ig loci, leaving the rest of the genome largely untouched. We are yet to understand the processes that subvert normal DNA repair machineries and instead wield components of these pathways to promote recombination of DSBs that could be over 100 kb apart. The molecular basis underlying the balance between normal and aberrant repair requires further elucidation. Such basic knowledge can be exploited to shift the fulcrum of repair judiciously in clinical settings of patients with immunodeficiency or lymphoid malignancies to reap translational benefit. Finally, DSB response occurs in, and is strongly influenced by the chromatin microenvironment. The dynamics of chromatin compaction and relaxation at DSBs are just beginning to unravel (140), but clearly much more remains to be unearthed as to how the dynamics of histone and DNA modifications impact and regulate a programed DSB response that ensues during AID-orchestrated CSR. Addressing these exciting issues will be at the forefront of research in the coming years.

## Conflict of Interest Statement

The authors declare that the research was conducted in the absence of any commercial or financial relationships that could be construed as a potential conflict of interest.
